# From Observation to Structured Reporting: Development and Preliminary Evaluation of an Online Animal Abuse Reporting System †

**DOI:** 10.3390/vetsci13080784

**Published:** 2026-08-05

**Authors:** Annamária Kiss, Gábor Lorászkó, Gabriella Korsós, Dorottya Bozsó, Zsombor Wagenhoffer, Kinga Fodor

**Affiliations:** 1 Department of Laboratory Animal Science and Animal Welfare, University of Veterinary MedicineBudapest, 1078 Budapest, Hungary; korsos.gabriella@univet.hu (G.K.); bozso.dorottya@univet.hu (D.B.); fodor.kinga@univet.hu (K.F.); 2 Department of Anatomy and Histology, University of Veterinary Medicine Budapest, 1078 Budapest, Hungary; loraszko.gabor@univet.hu; 3 Institute for Animal Breeding, Nutrition and Laboratory Animal Sciences, University of Veterinary Medicine Budapest, 1078 Budapest, Hungary; wagenhoffer.zsombor@univet.hu

**Keywords:** animal welfare, animal abuse, online reporting system, structured reporting, animal protection, veterinary assessment, decision support, digital animal welfare tool

## Abstract

Animal abuse and substandard animal care may be difficult to report when members of the public are uncertain about the seriousness of their observations, the competent authority, or the information needed for further assessment. This study aimed to develop and preliminarily evaluate a structured online reporting interface for suspected animal abuse and animal welfare concerns in Hungary. Two public online questionnaires assessed reporting-related knowledge, experiences, and preferences, and a separate professional questionnaire examined the experiences of actors involved in animal protection procedures. Based on these findings, the Hungarian legal context, and international reporting models, a browser-based prototype was developed and professionally tested by staff members of the National Food Chain Safety Office. Public respondents significantly differentiated between suspected criminal animal cruelty and substandard animal keeping: the police were selected more often for animal cruelty cases, whereas the notary was selected more often for substandard keeping cases (*p* < 0.001). Substandard keeping conditions were observed significantly more frequently than direct animal abuse (*p* < 0.001). Professional testing indicated that the prototype was understandable, transparent, and potentially useful, although further refinement, institutional integration, and field validation are required.

## Simple Summary

Suspected animal abuse and substandard animal care and housing conditions can be difficult to report because members of the public may be unsure how serious the situation is, what information should be recorded, or which authority should receive the report. Professionals also need clear and comparable information to assess cases and decide on the appropriate response. This study aimed to develop and preliminarily evaluate a structured online reporting interface for suspected animal abuse and animal welfare concerns in Hungary. The interface guides users through targeted questions about the animal, its environment, visible injuries, behaviour, risks, and available photographic or video evidence. It was designed to potentially support more accurate public reporting and to provide veterinarians, authorities, and animal welfare professionals with information that may be useful during preliminary case assessment or on-site inspections. The results showed that respondents partly distinguished between criminal animal cruelty and substandard animal keeping, but uncertainty about reporting pathways remained. Professional feedback indicated that the prototype was understandable and useful, although further technical and content-related refinement is needed before practical implementation.

## 1. Introduction

Animal welfare has become an increasingly important scientific, ethical, legal, and societal issue. In modern animal protection, animals are no longer considered only from the perspective of ownership or productivity, but as sentient beings whose physical condition, behaviour, environment, and mental state must be considered when assessing their welfare [1,2]. This broader view has increased the need for reliable methods that can help identify animal cruelty, neglect, and substandard animal care and housing conditions at an early stage.

In the present manuscript, animal abuse is used as a broad term covering human actions or omissions that cause, or may cause, harm, suffering, impaired welfare, or unnecessary risk to an animal. Active abuse refers mainly to harmful acts of commission, such as physical violence or deliberate injury, whereas neglect refers to failures of care, including inadequate provision of food, water, shelter, veterinary attention, hygiene, or appropriate living conditions. Substandard animal care and housing conditions are used here to describe keeping conditions that do not meet the animal’s welfare needs and may range from poor husbandry to legally relevant animal welfare infringements. These scientific working definitions are intended to clarify the terminology used in the manuscript and do not replace the national legal definitions discussed below.

The recognition of suspected animal abuse is complex. It may involve visible injuries, abnormal behaviour, inadequate nutrition or water supply, inappropriate housing, restricted movement, lack of veterinary care, environmental hazards, or inconsistencies between the reported history and the animal’s condition [3,4,5]. However, the interpretation of these signs is not always straightforward. A single indicator is rarely sufficient to confirm cruelty, while several findings considered together may raise concern and justify further professional or legal assessment [4,5]. This is particularly important because animal welfare cases are often first observed by laypersons, such as neighbours, animal keepers, or animal protection volunteers, who may not have the knowledge or confidence to evaluate the severity of a situation or decide where and how to report it.

Official and forensic veterinarians have a central role in the identification and documentation of suspected animal abuse, including active cruelty and neglect. Their professional expertise allows them to assess injuries, clinical condition, pain, body condition, behaviour, and the potential consequences of inadequate care [3,5]. Beyond the individual animal protection case, veterinary recognition and documentation may also be relevant because violence against animals has been described as being associated with violence against humans, including interpersonal and family violence [6]. Animal protection cases are frequently handled by several actors, including veterinarians, animal welfare organisations, local authorities, police, and administrative authorities. In Hungary, this multi-actor structure is further shaped by the parallel but procedurally distinct administrative and criminal legal frameworks. The Act on the Protection and Welfare of Animals provides the basis for administrative animal welfare proceedings, whereas Section 244 of the Criminal Code defines the criminal offence of animal cruelty [7,8]. Consequently, the same lay observation may require administrative action, criminal investigation, or both, depending on the nature and severity of the case. This legal and institutional complexity may improve case management when responsibilities are clear, but it can also contribute to uncertainty about competence, incomplete information transfer, and delays in intervention.

**Figure 1:**
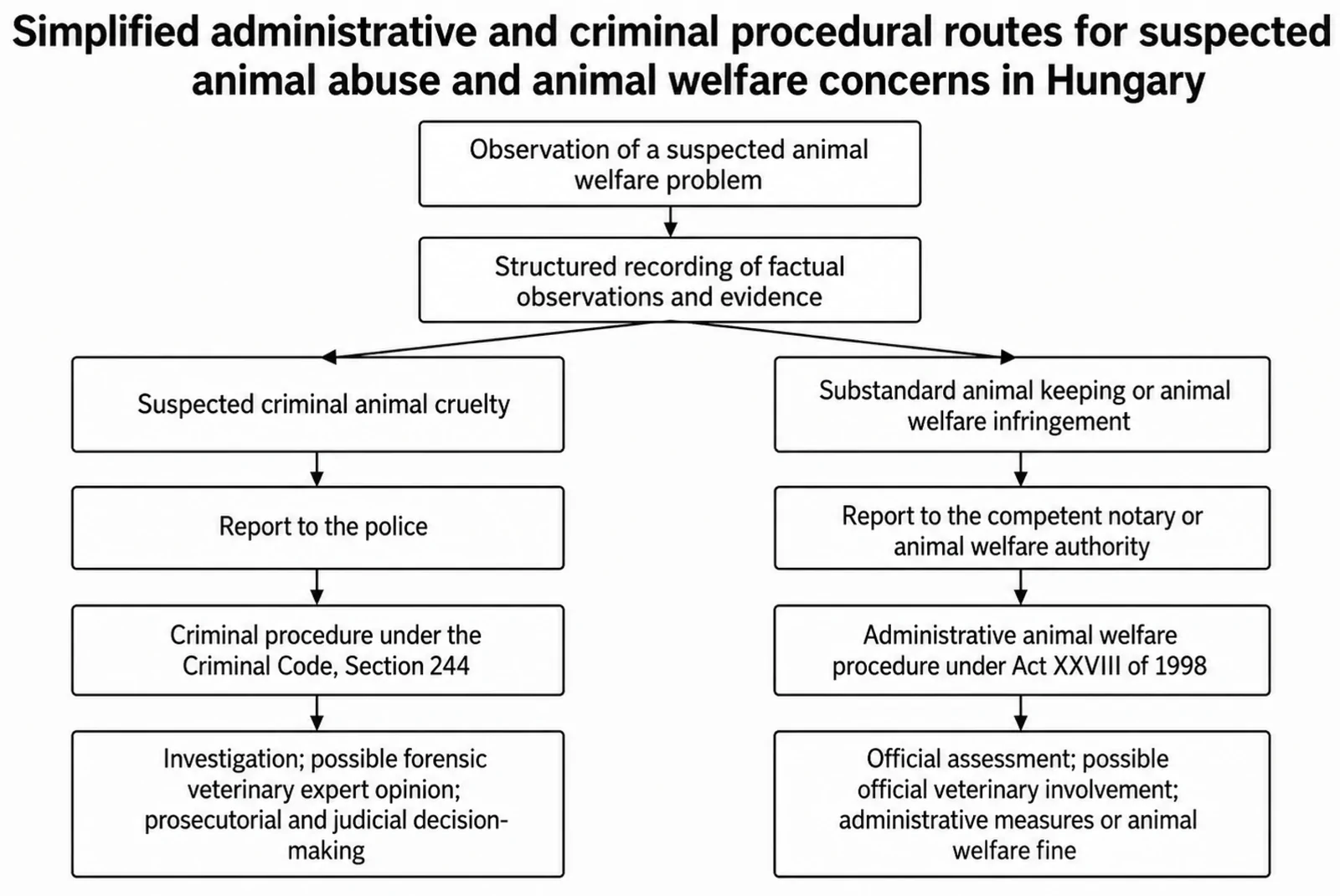
Simplified administrative and criminal procedural routes for suspected animal abuse and animal welfare concerns in Hungary. Cases involving suspected criminal animal cruelty should be reported to the police and may proceed under Section 244 of the Criminal Code. Cases involving substandard animal keeping, inadequate care, or broader animal welfare infringements may follow the administrative animal welfare pathway under Act XXVIII of 1998 on the Protection and Welfare of Animals. The two pathways are procedurally distinct but not necessarily mutually exclusive; where the facts of the case justify both administrative animal welfare measures and criminal investigation, the procedures may occur in parallel or consecutively. Structured factual reporting may support the competent authority in determining the appropriate procedural route. Figure created by the authors based on the Hungarian animal welfare and criminal legal framework [7,8].

In Hungary, the practical handling of reported cases depends on whether the situation primarily falls within an administrative animal welfare pathway or a criminal pathway (Table 1). Cases involving suspected criminal animal cruelty should be reported to the police, where a criminal procedure may be initiated if the conduct corresponds to the offence of animal cruelty under Section 244 of the Criminal Code [8]. By contrast, cases involving substandard animal keeping, inadequate care, or broader animal welfare infringements may be handled through the administrative animal protection pathway, most commonly by the competent municipal notary (in Hungary, a senior local administrative official with public authority functions, not a civil-law notary or notary public), by a local administrative authority, or by another animal welfare authority under the Act on the Protection and Welfare of Animals [7]. This distinction is important because the two legal frameworks overlap in everyday perception but differ in their procedural logic, competent authority, evidentiary requirements, and possible legal consequences (Figure 1). Therefore, a lay observer may not be able to determine whether a case should be submitted as a criminal complaint, an administrative animal welfare report, or both.

The need for clearer reporting pathways is also supported by comparison with the animal abuse reporting options available in countries neighbouring Hungary (Table 2). Although these countries differ in their legal and institutional structures, their systems show several recurring models, including emergency police reporting, veterinary or administrative inspection, online complaint forms, animal welfare ombudspersons, and civil legal or practical support for reporters [9,10,11,12,13,14,15]. Slovenia provides a particularly transparent example of state-based electronic reporting, including the possibility of anonymous submission to the competent food safety and veterinary authority [15]. Slovakia applies a structured online submission system linked to the State Veterinary and Food Administration and its regional bodies [10]. Austria relies on a decentralised provincial system, supported by animal welfare ombudspersons, while urgent cases may be directed to the police [9]. Romania operates through the Animal Police, especially when an animal is in immediate danger [12]. Serbia and Croatia illustrate more inspection- or authority-centred models, whereas Ukraine demonstrates the complementary role of civil legal assistance in preparing police reports [11,13,14]. These international examples indicate that effective reporting requires accessible entry points, clear identification of the competent authority, separation of urgent and non-urgent cases, and the possibility of attaching factual evidence. They also support the rationale for a Hungarian interface that does not require lay reporters to decide in advance whether an observed case should be treated primarily as an administrative, criminal, or general animal welfare matter.

The practical relevance of animal abuse reporting in Hungary is supported by previous police-based research. A national study involving 99 police stations examined 1169 animal-cruelty-related cases recorded between July 2013 and December 2021 and reported that official proceedings were initiated in a substantial proportion of reports [16]. These data indicate that suspected animal abuse is not only an animal welfare issue but also a matter of legal, professional, and public relevance. The reporting of suspected animal abuse also has broader public health and social significance. Previous studies have described associations between animal abuse and interpersonal violence, including domestic violence, child abuse, and elder abuse [17,18,19]. Animal abuse may therefore serve as a warning sign of wider household or community-level risk, and better documentation of such cases may support both animal welfare and broader violence-prevention research. This perspective is consistent with the One Welfare approach, which emphasises the interconnections between animal welfare, human well-being, and the wider social environment [20].

Despite the increasing recognition of these links, the practical reporting of suspected animal abuse remains challenging. Reports may be incomplete, emotionally framed, insufficiently documented, or directed to an inappropriate organisation. Members of the public may fail to report because they are uncertain whether the observed situation is serious enough, fear retaliation or neighbourhood conflict, do not know which authority is competent, or are unable to collect evidence that can be used in subsequent professional, administrative, or criminal evaluation [5,21,22]. Conversely, some reports may concern situations that do not require urgent intervention or do not fall within the competence of the contacted organisation. Such uncertainty can consume professional time and resources and may delay the recognition of cases in which rapid intervention is necessary.

Structured reporting tools may help address these problems. Digital technologies are increasingly used in animal welfare assessment, monitoring, data collection, and decision support [23]. However, much of the current literature focuses on production systems, sensor-based monitoring, or automated welfare indicators, while less attention has been paid to digital tools that guide citizens and professionals in reporting suspected animal abuse [22,24]. A structured online reporting interface may offer several advantages: it can guide the observer through relevant questions, reduce reliance on unstructured free-text descriptions, improve the comparability of reports, support the collection of photographic or video evidence, and provide professionals with more consistent information for preliminary case assessment. Such a system may also be useful during on-site inspections, where standardised data collection can support later expert evaluation and official procedures.

At the same time, structured reporting systems must be carefully designed. A tool that is too simple may fail to collect information needed for professional assessment, whereas an overly detailed or technical questionnaire may discourage lay users or lead to inaccurate responses. This creates a practical tension between accessibility and professional usefulness. Therefore, the development of an online animal abuse reporting interface should consider both the needs of the public and the requirements of veterinarians, authorities, and animal protection professionals.

Although complaint submission options and authority portals exist in several countries, most systems primarily provide an entry point for submitting concerns rather than an empirically informed, species- and context-specific intake structure that translates lay observations into professionally interpretable animal welfare information. In this context, a structured reporting interface may contribute not only as a technical reporting channel, but also as a methodological framework for organising lay observations before professional assessment. The present study therefore combines public and professional questionnaire data, legal and procedural analysis, comparison with neighbouring reporting models, prototype development, and preliminary professional evaluation. This approach allows the reporting process to be examined from both the reporter’s and the professional evaluator’s perspective, and helps identify which types of information should be collected before subsequent veterinary, administrative, or criminal assessment. The aim of the present study was to develop and preliminarily evaluate a structured online reporting interface for suspected animal abuse and animal welfare concerns in Hungary, primarily for use by non-expert public reporters, such as animal keepers, neighbours, witnesses, and animal protection volunteers, while also providing structured information that may support veterinarians, official veterinarians, authorities, and animal protection professionals during preliminary case assessment. The specific objectives were to identify practical problems in the reporting process, develop a question-based interface that supports the collection of relevant animal welfare information, and assess the professional usability of the prototype. The interface was designed to guide users through species- and situation-specific questions concerning the animal, its environment, visible injuries, behaviour, potential risks, and available visual documentation. The principal conclusion of the study is that a structured digital reporting system may reduce uncertainty among reporters, improve the quality and comparability of submitted information, support preliminary severity assessment, and assist professionals involved in animal protection procedures. Further technical refinement, institutional integration, and controlled field validation are required before routine implementation.

## 2. Materials and Methods

### 2.1. Study Design

The study was designed as a questionnaire-informed development and preliminary professional evaluation of a structured online reporting interface for suspected animal abuse and animal welfare concerns in Hungary. The work consisted of four consecutive phases: (1) assessment of reporting-related knowledge, experiences, and preferences among members of the public; (2) assessment of professionals’ experiences with the receipt, content, perceived reliability, emotional burden, duration, and procedural handling of animal protection reports; (3) development of a structured online reporting interface based on the questionnaire findings, the Hungarian legal and procedural context, and the animal welfare literature; and (4) preliminary professional testing of the prototype by staff members of the National Food Chain Safety Office.

The system was developed for the Hungarian animal protection context, in which suspected cases may require administrative animal welfare proceedings, criminal proceedings, or both, depending on the nature and severity of the case [7,8]. The aim of the study was not to validate the system under live reporting conditions, but to develop and preliminarily evaluate a professionally usable prototype capable of collecting structured information for subsequent case assessment.

### 2.2. Public Questionnaires

Two self-administered online questionnaires were created using Google Forms and distributed between 10 December 2024 and 10 June 2025. The questionnaires were shared through online platforms and social media groups related to animal keeping and animal protection. The target population included individuals interested in or affected by animal welfare and animal protection issues. Participation was voluntary. The recruitment channels were selected purposively in order to reach respondents likely to encounter, recognise, or report animal welfare-related situations, including animal keepers, animal protection volunteers, and individuals interested in animal protection. This recruitment strategy was appropriate for the exploratory aim of the study, but it was not intended to produce a representative sample of the Hungarian general population. Therefore, the public questionnaire findings should be interpreted as describing the sampled respondents rather than Hungarian society as a whole. The questionnaires were prepared in Hungarian. Respondents were not restricted by geolocation or residence verification. Responses were collected anonymously, and no names, email addresses, or other direct personal identifiers were requested. In order to maintain anonymity, no IP-based, login-based, or device-based duplicate-prevention method was applied; therefore, duplicate completion cannot be fully excluded. Demographic characteristics were described descriptively and were not compared with national population data because the study used a voluntary, non-probability exploratory sample.

The first questionnaire consisted of eight questions and assessed respondents’ knowledge of animal protection procedures, their understanding of the competent actors in cases of animal abuse and substandard animal keeping, and their preferences regarding possible reporting channels. The questionnaire also asked whether respondents would use a reporting option designed to support the clarification and documentation of animal protection cases. A total of 169 responses were received.

The second questionnaire consisted of 15 questions and focused on respondents’ own experiences with observing suspected animal abuse or substandard animal keeping conditions. The questionnaire followed a stepwise model of the reporting process, including observation, action, submission of a report, reporting method, and recipient of the report. It also asked whether any feedback or action was known to have followed the observed case. A total of 238 responses were received, providing the larger dataset on public experiences with animal protection-related situations.

The public questionnaires were designed to be short, clear, and suitable for completion on mobile devices. Questions were worded in simple terms and contained only a limited number of open-text fields in order to reduce respondent burden and support completion.

### 2.3. Professional Questionnaire

A separate online questionnaire was created using Google Forms and distributed among individuals professionally involved in animal protection procedures between 5 January 2025 and 30 March 2025. The questionnaire was shared through professional online groups and mailing lists. A total of 52 respondents completed the eight-question survey.

The questionnaire assessed the respondent’s role in animal protection cases, the channels through which reports are usually received, the perceived proportion of substantiated reports, the emotional burden associated with such cases, the typical duration of ongoing procedures, and factors that could accelerate case handling. It also assessed professional support for the development of a unified online reporting interface.

Responses were used to identify procedural and professional requirements for a reporting system, including the need for clearer routing, structured data collection, improved evidentiary support, veterinary involvement, and more efficient preliminary case assessment.

### 2.4. Development of the Online Reporting Interface

The online reporting interface was developed on the basis of the public and professional questionnaire findings, the literature review of animal welfare and animal protection procedures, the Hungarian administrative and criminal legal context, and the comparison of reporting pathways in countries neighbouring Hungary. The aim of the interface was to guide non-expert reporters through structured, observation-based questions while collecting information that may be useful for professionals, authorities, and official or forensic veterinarians.

An earlier conference proceeding presented the initial concept and development rationale of the online reporting interface; the present study extends that work by integrating questionnaire-based findings, the Hungarian legal and procedural context, comparison with neighbouring countries, and preliminary professional prototype evaluation [25].

The system was designed as a browser-based web interface rather than as a mobile application. This design choice was intended to avoid the need for downloading, installing, or updating an application, which may represent a barrier for older users or individuals with lower digital confidence. The interface could be accessed from desktop computers and mobile devices with internet access.

The reporting workflow was organised into separate pathways according to the type and context of animal keeping. This structure was chosen because different animal-keeping situations require different information for preliminary assessment. Instead of using a single general reporting form, the interface directed the user to a situation-specific set of questions. The detailed content of these pathways is presented in Section 3 and in the interface structure table.

The interface was developed in Hungarian with external technical assistance for web development. The wording of the questions was designed to remain understandable for lay users while still collecting information relevant to professional assessment. The system did not assign legal conclusions, veterinary diagnoses, or final welfare ratings. Instead, it was designed to record observable facts and supporting evidence that may assist later professional, administrative, or criminal evaluation.

A minimum information framework was defined during development to support the preliminary usability of submitted reports. This framework included sufficient information to identify the type of case, locate the observation, support initial credibility assessment, and determine whether further professional or official action may be required. The purpose of the framework was to assess whether a submitted report contains enough information for preliminary severity assessment, categorisation, and selection of the possible further procedural pathway.

### 2.5. Preliminary Professional Testing of the Prototype

After the development of the online interface, preliminary testing was conducted between 1 April 2025 and 1 June 2025. The prototype was sent to staff members of the National Food Chain Safety Office for professional evaluation. A total of 24 staff members participated in the testing process.

The evaluation should be interpreted as structured professional expert feedback rather than as a formal usability study or direct lay-user usability testing. The feedback on lay-user accessibility therefore reflects the professional judgement of the testers and not the direct experience of non-expert public users. Testers were asked to navigate the prototype reporting pathways and provide written feedback on the clarity of the website, comprehensibility of the questions, appropriateness of the reporting categories, and technical functioning of the interface. No validated usability instrument was used, and no quantitative usability metrics were recorded. Testers were not blinded to the general purpose of the prototype, as the aim was to obtain professional feedback on a proposed animal protection reporting tool. The written comments were reviewed qualitatively and grouped into technical issues, question comprehensibility, lay-user accessibility, reporting structure, and procedural information needs. The individual professional backgrounds of the testers were not analysed systematically; therefore, the findings are reported as institutional expert feedback from staff members of the National Food Chain Safety Office. The testing focused on professional and user-oriented evaluation of the prototype rather than the processing of real animal protection reports.

The prototype was not opened for live public reporting during the study period. At that stage, the system did not include a central back-end module for the official receipt, storage, professional pre-screening, and forwarding of submitted reports. For this reason, and because it would not have been ethically appropriate to record real cases requiring intervention solely for testing purposes, the evaluation was limited to controlled professional review.

### 2.6. Data Processing and Statistical Analysis

Questionnaire data were exported from Google Forms and prepared for analysis using Microsoft Excel. Statistical analyses were performed using R Commander, version 4.6.0. Categorical variables are summarised as counts and percentages. For questions allowing multiple answers, each response category was transformed into a separate binary variable; therefore, percentages for multiple-choice questions could exceed 100%. Denominators vary where respondents did not answer a given question.

For the first questionnaire, paired nominal data were analysed using McNemar’s test to compare respondents’ selection of competent actors for animal cruelty cases and substandard animal keeping cases. A composite digital reporting variable was created for respondents who selected at least one digital reporting channel, such as email, an online interface, or an application. Support for a structured online reporting system was also assessed using a score ranging from 0 to 3, based on the selection of guided questions, image-upload options, and rapid reporting availability at the place of observation.

For the second questionnaire, open-ended responses were analysed by content analysis. Recurrent themes were identified, grouped into main categories, and quantified. The purpose of the qualitative analysis was to identify barriers, uncertainties, and recurring problems reported by respondents in relation to animal protection reporting. Paired nominal data were compared using McNemar’s test; when sample size or the distribution of discordant pairs required it, exact McNemar’s test was applied. Discordant pairs and paired odds ratios were reported where relevant.

For the professional questionnaire, composite indicators were created for digital reporting channels, the perceived need for veterinary involvement, procedure duration shorter or longer than three months, and support for a unified reporting interface. One-sample exact binomial tests were used to examine whether selected proportions significantly exceeded 50%. Proportions were reported with counts, percentages, and 95% confidence intervals. Associations between categorical variables were assessed using chi-square tests or Fisher’s exact tests when expected cell counts were low. For multi-category variables, the strength of association was described using Cramer’s V. For 2 × 2 comparisons, odds ratios and 95% confidence intervals were calculated. The threshold for statistical significance was set at *p* < 0.05.

### 2.7. Ethical Considerations

The study did not involve experimental animals, clinical animal patients, or intervention in live animal protection cases. The questionnaire surveys involved respondents and were conducted anonymously. The preliminary prototype evaluation was performed as professional feedback on the usability and content of the reporting interface. No personal identifying data are reported in this manuscript.

Ethical review and approval were waived for this study because the questionnaires and professional feedback were anonymous, voluntary, non-interventional, and did not involve the processing of sensitive personal data. Informed consent was considered to be provided by voluntary completion and submission of the questionnaires or by written professional feedback.

### 2.8. Use of Generative Artificial Intelligence

No generative artificial intelligence tool was used for data collection, questionnaire analysis, statistical testing, interpretation of results, or development of the reporting interface.

## 3. Results

### 3.1. Public Knowledge of Animal Protection Procedures and Reporting Preferences

The first public questionnaire received 169 responses. The 18–35-year and 36–65-year age groups were equally represented, with 82 respondents in each group (48.5% each), while 5 respondents (3.0%) were older than 65 years. Slightly more than half of the respondents were female (*n* = 91; 53.8%), and 78 respondents were male (46.2%).

When respondents were asked which actors they considered officially responsible for handling animal cruelty cases, the police were selected most frequently (*n* = 137; 81.1%). Other frequently selected actors were the notary (*n* = 82; 48.5%), the official veterinarian (*n* = 77; 45.6%), and animal welfare organisations (*n* = 74; 43.8%). Fewer respondents selected the private veterinary practitioner (*n* = 26; 15.4%), government offices (*n* = 21; 12.4%), the National Food Chain Safety Office (*n* = 18; 10.7%), or “do not know” (*n* = 5; 3.0%).

A different pattern emerged for substandard animal care and housing conditions. In this case, respondents most frequently selected the notary (*n* = 102; 60.4%), followed by the police (*n* = 86; 50.9%), the official veterinarian (*n* = 77; 45.6%), animal welfare organisations (*n* = 68; 40.2%), the private veterinary practitioner (*n* = 34; 20.1%), the National Food Chain Safety Office (*n* = 24; 14.2%), government offices (*n* = 23; 13.6%), and “do not know” (*n* = 9; 5.3%). This difference indicates that lay respondents partly distinguished between suspected criminal animal cruelty and substandard animal keeping, but their answers also demonstrated uncertainty regarding the division of responsibilities among the competent actors. In the paired comparison, the police were selected significantly more often for animal cruelty cases, whereas the notary was selected significantly more often for substandard animal keeping cases (*p* < 0.001 for both comparisons). This comparison reflects respondents’ perceived reporting choices rather than a legally or administratively verified classification of the cases.

Regarding preferred reporting channels, telephone reporting was selected most frequently (*n* = 112; 66.3%), followed by online reporting (*n* = 74; 43.8%), email (*n* = 60; 35.5%), and application-based reporting (*n* = 58; 34.3%). Overall, 118 respondents (69.8%) selected at least one digital reporting option, including email, an online interface, or an application.

Respondents also supported functions that could improve the quality and usability of animal protection reports. The most frequently selected option for filtering false, inaccurate, or insufficient reports was the possibility of uploading photographs (*n* = 112; 66.3%). This was followed by a rapidly accessible reporting option at the place of observation (*n* = 87; 51.5%) and the use of guided questions (*n* = 83; 49.1%). A composite support score was calculated from three functions: guided questions, image upload, and rapid on-site reporting. Only 5 respondents (3.0%) selected none of these functions, whereas 73 respondents (43.2%) selected one, 64 respondents (37.9%) selected two, and 27 respondents (16.0%) selected all three. Overall, 164 respondents (97.0%) selected at least one function supporting a structured online reporting system, and 91 respondents (53.8%) selected at least two.

### 3.2. Public Experiences with Observing and Reporting Animal Abuse and Substandard Keeping Conditions

The second public questionnaire received 238 responses. The largest age group was the 36–65-year group (*n* = 124; 52.1%), followed by respondents aged 18–35 years (*n* = 109; 45.8%). Five respondents (2.1%) were older than 65 years. Most respondents were female (*n* = 156; 65.5%), 81 respondents were male (34.0%), and one respondent did not wish to answer this question (0.4%).

A total of 34 respondents (14.3%) reported that they had directly observed a case that they considered animal abuse within the previous three years. In comparison, 115 respondents (48.3%) reported observing animals kept under substandard conditions. At least one of these two animal protection problems was observed by 121 respondents (50.8%), while 28 respondents (11.8%) had observed both animal abuse and substandard keeping conditions. Substandard keeping conditions were observed significantly more often than direct animal abuse (McNemar χ^2^ = 68.82, df = 1, *p* < 0.001).

The open-ended descriptions of observed animal abuse cases were grouped into four main categories. Active abuse, including hitting, kicking, or pulling, was the most frequently described category (12 mentions). Nutrition-related problems, such as starvation, water deprivation, or marked thinness, were mentioned in 9 cases. Housing-related problems, including permanent tethering, overcrowding, or lack of shelter, were mentioned in 5 cases. Lack of care for disease or injury was mentioned in 1 case. Some responses contained more than one type of problem and were therefore considered in more than one category.

Among respondents who believed they had observed animal abuse, the most frequent reaction was to address the person involved without submitting an official report (*n* = 11; 32.4%). A further 10 respondents (29.4%) reported both expressing their objection to the person involved and submitting a report, while 4 respondents (11.8%) reported the case without personal confrontation. Three respondents (8.8%) did not take any action. Among respondents who provided a reason for not reporting the observed animal abuse (*n* = 13), the most frequently selected reason was not knowing where or to whom the report should be submitted (*n* = 5; 38.5%). Other reasons included uncertainty about what would happen to the reported person (*n* = 3; 23.1%), concern about the future of the animal (*n* = 2; 15.4%), fear of further consequences (*n* = 2; 15.4%), and inability to prove what had been observed (*n* = 1; 7.7%).

A similar pattern of reporting barriers was found among respondents who observed substandard keeping conditions. Among respondents who provided a reason for not reporting such conditions (*n* = 65), the most frequent reasons were concern about the future of the animal (*n* = 26; 40.0%) and not knowing where or to whom the report should be submitted (*n* = 25; 38.5%). Other reasons included uncertainty about what would happen to the reported person (*n* = 13; 20.0%), fear of further consequences (*n* = 11; 16.9%), and lack of interest in the situation (*n* = 4; 6.2%).

Among responses containing a report recipient for substandard keeping conditions (*n* = 42), animal welfare organisations were contacted most frequently (*n* = 19; 45.2%), followed by the notary and the municipality (*n* = 18 each; 42.9%), the police (*n* = 10; 23.8%), a public online platform (*n* = 4; 9.5%), the National Food Chain Safety Office (*n* = 1; 2.4%), and the district veterinary authority (*n* = 1; 2.4%). When report recipients were grouped into broader categories, 31 respondents (73.8%) had contacted at least one official or authority-related route, while 19 respondents (45.2%) had sought help from a civil animal welfare organisation.

Within the sampled respondents, these findings suggest that the first barrier was not necessarily the recognition of a possible animal welfare problem, but the transition from observation to a complete, correctly routed, and usable report. Lack of knowledge about the competent authority, uncertainty about the consequences of reporting, concern about the animal’s future, and difficulty in providing evidence were recurring barriers in both animal abuse and substandard keeping cases.

### 3.3. Professional Experiences with Animal Protection Reports

The professional questionnaire was completed by 52 respondents actively involved in animal protection procedures. The largest respondent groups were notaries (40.4%) and civil animal protection workers (30.8%). Other respondents included police officers, notary clerks, administrative officers, official authority staff, and animal control facility staff.

Reports were most frequently received by email (67.3%) and telephone (63.5%). Personal reports were also common (40.4%), while online submissions were less frequent (25.0%) and application-based submissions were rare (1.9%). At least one digital channel, defined as email, online submission, or application-based reporting, was selected by 43 respondents (82.7%; 95% CI: 69.7–91.8%). This proportion was significantly higher than 50% (*p* < 0.001). In the paired comparisons, email and telephone reporting did not differ significantly from each other (67.3% vs. 63.5%; paired OR = 1.20; *p* = 0.832), but both were significantly more frequent than online reporting (email vs. online: paired OR = 3.75, *p* < 0.001; telephone vs. online: paired OR = 6.00, *p* < 0.001).

Respondents were also asked to estimate the proportion of incoming reports that represented genuine animal protection cases. The largest group of respondents (35.3%) estimated that 60–80% of incoming reports were genuine cases requiring animal protection procedures. Overall, 26 of 51 respondents (51.0%; 95% CI: 36.6–65.2%) estimated that more than 60% of incoming reports were genuine animal protection cases. This proportion did not significantly exceed 50% (*p* = 0.500), indicating variability in professional experience regarding the reliability and relevance of incoming reports.

Animal protection procedures were also associated with emotional burden. Half of the respondents considered the emotional burden of such cases to be case-dependent, while 34.6% reported that these cases were emotionally burdensome. In contrast, 9.6% did not consider them emotionally burdensome.

A high proportion of professional respondents supported the involvement of veterinary expertise. After combining response categories, 46 of 51 respondents (90.2%; 95% CI: 78.6–96.7%) considered the involvement of a veterinarian necessary for assessing the animal’s condition. This proportion was significantly higher than 50% (*p* < 0.001). Although 63.5% of respondents considered themselves competent in the professional assessment of animal protection cases, the need for veterinary involvement remained high, indicating that structured reporting should support, but not replace, professional veterinary or official evaluation.

Most respondents reported that animal protection cases were usually completed within three months. The most frequent category was 1–3 months (50.0%), followed by less than 1 month (21.2%), more than 12 months (13.5%), 6–12 months (9.6%), and 3–6 months (5.8%). After combining categories, 37 of 52 respondents (71.2%; 95% CI: 56.9–82.9%) indicated that cases were typically completed within three months, which was significantly higher than 50% (*p* = 0.002).

Support for a unified citizen reporting interface was present among professional respondents. In total, 65.4% indicated that such an interface would support the handling of animal protection cases (*p* = 0.018). Support was particularly high among animal protection workers (87.5%) and among respondents who experienced procedures lasting more than three months. Respondents reporting procedure durations longer than three months supported the unified reporting interface more frequently than those reporting procedures usually completed within three months (93.3% vs. 54.1%; OR = 11.90; 95% CI: 1.42–100.07; *p* = 0.009). These results suggest that the perceived usefulness of a structured interface increases where current procedures are experienced as longer or more burdensome.

### 3.4. Structure and Functional Output of the Online Reporting Interface

Based on the questionnaire findings, literature review, Hungarian legal context, and comparison with neighbouring countries, a structured online reporting interface was developed. The interface was designed as a Hungarian-language, browser-based system that guides the reporter through predefined categories and observation-based questions. Its purpose was not to establish a legal conclusion or veterinary diagnosis, but to collect factual information that can support preliminary case classification, professional review, and subsequent administrative or criminal routing.

**Figure 2:**
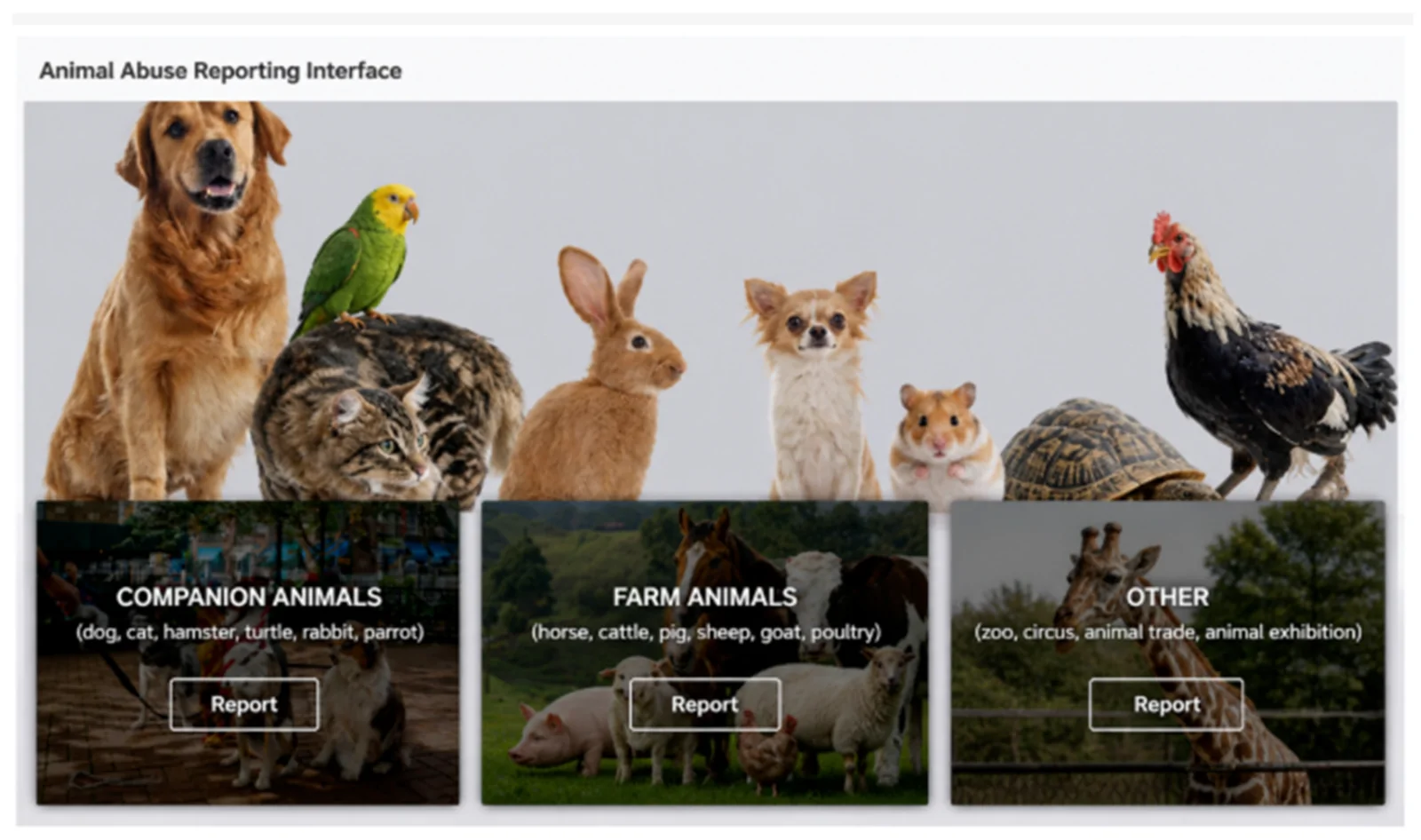
Homepage of the developed animal abuse reporting interface, illustrating the three principal entry categories: companion animals, farm animals and other contexts, including animals involved in entertainment, exhibition or trade. The operational interface was developed in Hungarian; the visible labels in the figure were translated into English for presentation and manuscript purposes. (Screenshot from the authors’ reporting interface).

The homepage separated the reporting process into three principal pathways: companion animals, farm animals, and other contexts (Figure 2). Within the companion animal pathway, separate routes were created for individually kept animals and group housing. The “other” category was intended for less typical but animal protection-relevant settings, such as zoos, circuses, pet shops, animal markets, exhibitions, or trade-related situations (Table 3). An example of the structure of the companion animal reporting pathway is provided in Supplementary Materials to illustrate how the prototype guides the reporter from general case information to animal-related observations, environmental conditions, urgency-related factors, and evidence upload.

The interface used primarily predefined answer options and only a limited number of free-text fields. This design was intended to reduce unnecessary narrative information, improve comparability between reports, and help lay users record observable facts rather than legal or veterinary conclusions. The guided questions were designed to assist both the reporter and the professional evaluator: the reporter is prompted to notice relevant welfare-related signs, while the receiving professional can review the submitted information in a more standardised format.

The functional structure of the interface directly reflected the main needs identified in the questionnaires. Public respondents supported photograph upload, rapidly accessible reporting, and guided questions. Professional respondents indicated that incoming reports often arrive through partly digital but unstructured channels, and they highlighted the need for complete, interpretable, and professionally usable information. The resulting prototype therefore represents a structured intake concept between lay observation and professional case assessment.

### 3.5. Preliminary Professional Evaluation of the Prototype

The prototype was evaluated by staff members of the National Food Chain Safety Office. The feedback was generally positive: testers described the interface as visually clear, transparent, easy to use, and professionally useful. Several testers indicated that they would use such a service in justified cases and considered the operation of a structured animal abuse reporting system necessary.

The evaluation also identified technical and content-related areas requiring further development. Technical issues included error messages during form submission, especially when testing the farm animal pathway, and loss of previously entered data after page refresh. Testers suggested clearer error messages and visual highlighting of incorrect or incomplete fields.

Content-related feedback mainly concerned the accessibility of the questions for lay users. Some questions were considered potentially difficult for non-expert reporters, for example questions requiring the assessment of animal behaviour or the size of the keeping area. Testers recommended reducing the number of questions and clarifying certain items. One suggested modification was to replace a question asking whether the animal appeared thin or obese with a broader body condition question that also allows the reporter to select normal or healthy condition. Testers also recommended adding explanatory text for cases in which an exact address cannot be provided, such as animals observed in public areas, along roads, or in rural outdoor locations. Additional development needs included a separate option for abandoned animals, recording whether temporary care had been arranged, whether animals had been removed from the location, and where they had been taken. Testers considered these details useful for determining the urgency and order of possible intervention. They also indicated the need for logical controls to prevent contradictory entries, such as inconsistent county, settlement, and address information or contradictory environmental conditions.

Overall, the professional evaluation indicated that the structured reporting approach was considered relevant and practically useful. The findings should be interpreted as the preliminary evaluation of a professionally reviewed prototype, not as validation of an operational public reporting system. Further development should focus on technical stability, simplification of the questionnaire, prevention of contradictory responses, and institutional integration before public implementation.

## 4. Discussion

The present study developed and preliminarily evaluated a structured online reporting interface for suspected animal abuse and animal welfare concerns in Hungary. Among the sampled respondents, the results support the initial working assumption that the reporting of animal welfare cases is not limited by public concern alone, but also by uncertainty regarding case interpretation, competent authorities, evidentiary requirements, and the practical steps of reporting. The findings also indicate that a structured digital interface may serve as a bridge between lay observation and professional case assessment by guiding the reporter toward factual, comparable, and professionally interpretable information.

A central finding was that respondents partly distinguished between suspected criminal animal cruelty and substandard animal keeping. The police were selected more frequently for animal cruelty cases, whereas the notary was selected more frequently for substandard animal keeping cases. This distinction is consistent with the Hungarian legal framework, in which serious animal cruelty may fall under criminal law, while broader animal welfare infringements and substandard keeping conditions are commonly addressed through administrative animal protection procedures [7,8]. However, this distinction should be interpreted cautiously, because the questionnaire measured respondents’ perceived classification and preferred reporting route rather than the actual outcome of administrative or criminal proceedings. In real cases, legal classification depends on subsequent fact-finding, authority assessment, and, where applicable, judicial decision-making supported by technical or veterinary forensic expertise. The simultaneous selection of several actors with different legal roles also indicates that lay understanding of the institutional system remains incomplete. This is practically important because reports submitted to an inappropriate body may require transfer, delay intervention, or result in incomplete case handling. These findings support the need for a reporting tool that does not require the reporter to make a legal classification in advance, but instead collects the factual elements needed for subsequent professional or authority-based routing.

The Hungarian context further strengthens the relevance of such a system. Previous police-based research showed that animal-cruelty-related reports in Hungary may lead to official proceedings in a substantial proportion of cases [16]. This indicates that public reports can have direct procedural consequences, provided that they contain information that is sufficiently complete, locatable, and interpretable. The proposed reporting interface was designed to address this need by shifting the focus from unstructured narrative reporting to guided observation-based reporting. This approach is also consistent with veterinary forensic recommendations, which emphasise the importance of accurate documentation, preservation of evidence, and systematic recording of clinical and contextual findings in suspected animal abuse cases [3,5].

In the present exploratory sample, observation of a possible animal welfare problem did not necessarily lead to a report. Respondents described barriers such as not knowing where to report, uncertainty about the consequences of reporting, concern about the animal’s future, and difficulty in proving what had been observed. These barriers correspond with previous work describing challenges in animal abuse investigations and the practical difficulty of obtaining reports that are actionable, objective, and legally useful [21]. They also reflect a broader problem in digital and institutional reporting systems: accessible reporting options are only effective if users understand what information is required and trust that the submission pathway is appropriate [22]. Therefore, the potential value of a structured online interface lies not only in digitalisation itself, but in the combination of accessibility, standardisation, and guidance.

The results from professionals involved in animal protection procedures further support this interpretation. Most professional respondents received reports through partly digital channels, especially email, but these channels do not necessarily ensure completeness, comparability, or appropriate evidentiary value. The high level of support for veterinary involvement confirms that a reporting interface should not replace professional evaluation. Rather, it should support veterinarians, official veterinarians, authorities, and animal protection professionals by improving the quality of the initial information available for assessment. This distinction is important because the interface was not designed to determine whether abuse had occurred, to assign legal responsibility, or to produce a veterinary diagnosis. Its function is to structure the first observation and provide a better starting point for subsequent professional and legal evaluation.

The comparison with reporting options in countries neighbouring Hungary also supports the rationale for a structured national reporting interface. The reviewed systems show different institutional models, including police-based emergency reporting, veterinary or administrative inspection, online complaint forms, animal welfare ombudspersons, and civil legal assistance [9,10,11,12,13,14,15]. These examples indicate that effective reporting systems require both accessible entry points and clear procedural routing. The proposed Hungarian interface could therefore contribute to a more transparent reporting pathway by collecting the information required for administrative or criminal handling without expecting the reporter to understand the full legal framework.

The structure of the developed prototype reflects this dual purpose. The three main reporting pathways—companion animals, farm animals, and other contexts—allow the system to adapt the reporting process to different animal-keeping situations. This is important because welfare concerns in a household companion animal setting may differ substantially from those in farm animal, trade, exhibition, or entertainment contexts. At the same time, the use of predefined answers and limited free-text fields may reduce emotionally framed or subjective reports and improve comparability between submissions. This design choice is consistent with the broader use of digital technologies in animal welfare assessment, monitoring, data collection, and decision-support processes [23]. However, unlike many digital animal welfare tools that focus on production systems or sensor-based monitoring, the present interface focuses on citizen-to-authority reporting and the translation of lay observations into professionally usable information.

Professional testing of the prototype provided encouraging but preliminary results. Testers considered the interface understandable, transparent, easy to use, and professionally useful. These findings support the practical relevance of the concept and suggest that such a system may be acceptable to professionals involved in animal protection work. However, the testing also identified several areas requiring improvement, including technical stability, clearer error messages, prevention of data loss after page refresh, reduction in the number of questions, and clarification of items that may be difficult for lay users. These points highlight an important design tension: the system must collect enough information for professional assessment, while remaining simple enough for non-expert users in potentially stressful situations. In addition, because the prototype was tested by staff members of the National Food Chain Safety Office, the findings reflect an institutional and professional perspective. These testers may have greater familiarity with certain types of animal protection cases, species, authority procedures, or evidentiary requirements than the general public. Consequently, their assessment of question clarity and lay-user accessibility may not fully capture the difficulties experienced by non-expert reporters in stressful real-life situations. Professional testers may identify important accessibility problems, but they may also underestimate or overestimate the cognitive burden, uncertainty, or practical barriers faced by lay users. Therefore, the results of the professional evaluation should not be extrapolated directly to the public target population, and the interface requires future testing with lay users before routine implementation.

Future development should therefore focus on reducing cognitive burden without reducing the professional value of the submitted information. A tiered questionnaire structure may be useful, in which all users first complete a short core module containing only essential observations, such as animal species, location, visible injuries, access to food and water, shelter, and immediate danger. More detailed questions could then appear only when relevant, using conditional branching based on the user’s previous answers. Questions requiring more subjective judgement, such as body condition, behaviour, pain, or the adequacy of the keeping area, should be supported by visual aids, pictograms, short examples, and explanatory tooltips. These aids should not be intended to replace veterinary assessment, but to help lay users describe observable signs more consistently. For example, simplified body condition illustrations could help reporters distinguish between apparently normal, underweight, and overweight animals without requiring formal veterinary knowledge. Similarly, behaviour-related questions could use observable descriptions, such as “unable to stand”, “continuous vocalisation”, “extreme fear”, or “unresponsive”, rather than technical behavioural terminology.

Logical controls should also be expanded to guide users during completion. These controls could include warning messages for missing essential information, automatic detection of contradictory answers, confirmation prompts for urgent cases, and context-sensitive suggestions when the user is uncertain. The interface could also include “I do not know” or “cannot assess” response options for questions that may exceed the reporter’s knowledge. This would help avoid inaccurate forced answers while still informing professionals which aspects require verification during follow-up or on-site inspection. In this way, the system could preserve professional utility while remaining accessible to non-expert users.

The findings also have broader social relevance. Animal abuse has been associated with interpersonal violence, including family violence and other forms of harm directed at humans [6,17,18,19]. Although animal abuse should not be interpreted as a direct predictor of human-directed violence in every case, improved documentation may contribute to a better understanding of these associations. This is consistent with the One Welfare approach, which emphasises the interconnections between animal welfare, human well-being, and the wider social environment [20]. A structured reporting system could therefore support not only individual animal protection cases, but also future research into patterns of animal abuse, household risk, and community-level welfare concerns.

Several limitations must be acknowledged. First, the public questionnaires were distributed online and relied on voluntary participation through animal keeping and animal protection-related channels. Therefore, the sample is unlikely to be representative of the general Hungarian population. Respondents may have had greater interest in animal welfare, higher awareness of reporting options, or stronger opinions about animal protection than the average citizen. Consequently, the findings on public knowledge, reporting preferences, and reporting behaviour should be interpreted as applying to the sampled respondents rather than to Hungarian society as a whole. Second, the questionnaires used predefined age categories rather than exact age values, which limits more detailed demographic interpretation. Third, the professional questionnaire included a relatively small and heterogeneous sample of actors involved in animal protection procedures. Although this diversity reflects the multi-actor nature of the field, subgroup analyses should be interpreted cautiously. Fourth, the prototype was evaluated through professional review rather than through live public reporting. Therefore, the present study cannot demonstrate that the interface improves reporting accuracy, accelerates intervention, reduces false reports, or improves legal outcomes in real cases.

Real-world implementation would require a data protection and governance framework before public launch. Reports may contain location data, photographs or videos, information about alleged offenders, and potentially identifiable household or property information. Therefore, the system would need clearly defined institutional responsibility, secure storage, restricted access rights, transparent information for users, appropriate retention periods, and procedures for handling anonymous and identified reports. These safeguards would be necessary both to protect reporters and reported persons and to preserve the evidentiary value of submitted material. Data protection should therefore be incorporated into the design of the reporting pathway from the beginning, rather than treated only as a technical issue after implementation.

The study also did not evaluate the downstream operation of a complete institutional case-management system. At the current stage, the interface should be interpreted as a professionally reviewed prototype and structured intake concept, not as a validated operational reporting system. Future implementation would require an institutional back-end capable of receiving, storing, pre-screening, and forwarding reports in accordance with legal, ethical, and data protection requirements. Particular attention should be paid to anonymous reporting, because it may reduce fear of retaliation but may also limit follow-up possibilities and evidentiary value. Before public implementation, the revised interface should be tested with lay users under controlled usability conditions. Future evaluation should measure completion time, user comprehension, frequency of missing or contradictory answers, perceived difficulty, willingness to complete the form, and the professional usability of the submitted reports. Particular attention should be paid to whether visual aids, tooltips, simplified wording, “I do not know/cannot assess” options, and conditional branching improve reporting quality without discouraging users from completing the form.

## 5. Conclusions

The present study developed and preliminarily evaluated a structured online reporting prototype for suspected animal abuse and animal welfare concerns in Hungary. Among the sampled respondents, uncertainty remained regarding competent authorities, reporting pathways, evidentiary requirements, and the practical steps of reporting. Professional feedback indicated that the structured approach was understandable and potentially useful, but also highlighted the need for technical refinement, questionnaire simplification, logical controls, institutional integration, and stronger data protection safeguards. The interface addresses these needs by guiding reporters through structured, observation-based questions and by supporting the collection of factual information and visual evidence. Its main value lies in translating lay observations into a format that may be more useful for veterinarians, authorities, and animal protection professionals. The system does not replace official, veterinary, or legal assessment; rather, it may provide a more reliable starting point for subsequent administrative or criminal procedures. The preliminary professional evaluation suggests that the structured reporting approach is relevant and practically useful. However, the prototype was not validated under real-world reporting conditions. Its potential effects on reporting accuracy, case prioritisation, intervention efficiency, documentation quality, and administrative or legal outcomes should therefore be evaluated in future field studies before routine implementation.

## Figures and Tables

**Table 1:** Main procedural and legal differences between administrative animal welfare cases and criminal animal cruelty cases in Hungary.

Aspect	Administrative Animal Welfare Pathway	Criminal Animal Cruelty Pathway
Main legal basis	Act XXVIII of 1998 on the Protection and Welfare of Animals	Act C of 2012 on the Criminal Code, Section 244
Typical cases	Substandard keeping, inadequate care, animal welfare infringements, neglect, housing or husbandry problems	Serious intentional conduct capable of causing permanent health damage or death, abandonment of vertebrate or dangerous animals, severe cruelty
Primary focus	The welfare, condition, and interests of the animal	The criminally relevant conduct of the offender
Competent route	Notary, animal welfare authority, official veterinary authority, or other administrative body depending on the case	Police and criminal justice authorities
Professional assessment	Official veterinary involvement may be part of the administrative assessment	Forensic veterinary expert opinion may be requested during the criminal procedure
Scope of animal protection	Broader welfare-based approach; includes physical and mental welfare concerns	Narrower criminal-law approach; applies to the most serious legally defined conduct

**Table 2:** Selected functional elements of animal abuse reporting systems in countries neighbouring Hungary.

Countries	Functional Element	Relevance for the Proposed Hungarian Interface
Austria	Animal welfare ombudsperson	Supports legally informed case handling
Slovakia	Structured online submission to a veterinary or food safety authority	Improves consistency of administrative assessment
Ukraine	Civil legal support and police-report templates	Helps prepare procedurally usable reports
Romania, Austria	Emergency police reporting	Distinguishes urgent cases from welfare concerns
Serbia	Veterinary or internal affairs reporting	Highlights the need for clear institutional routing
Croatia	Veterinary inspection-based handling	Emphasises factual, evidence-based submissions
Slovenia	Electronic and anonymous reporting to a competent authority	Lowers reporting barriers and supports accessibility

**Table 3:** Principal reporting pathways of the online animal abuse reporting interface.

Reporting Pathway	Intended Reporting Context	Rationale for Separation
Companion animals	Individually or group-kept companion animals	Care, housing, treatment, injury, behaviour, and neglect concerns
Farm animals	Small-scale or production-related farm animal keeping	Group-level housing, feeding, watering, hygiene, and welfare concerns
Other contexts	Zoos, circuses, pet shops, markets, exhibitions, trade, or transport	Welfare risks linked to use, display, trade, or non-standard keeping

## Data Availability

The original contributions presented in this study are included in the article/Supplementary Materials. Further inquiries can be directed to the corresponding author.

## Supplementary Materials

The following supporting information can be downloaded at https://www.mdpi.com/article/10.3390/vetsci13080784/s1, Supplementary Table S1—First Public Questionnaire; Supplementary Table S2—Second Public Questionnaire; Supplementary Table S3—Professional Questionnaire; Supplementary S4.
