# Supplementary material for: From Observation to Structured Reporting: Development and Preliminary Evaluation of an Online Animal Abuse Reporting System
Source: Vet Sci. 2026 Aug 5;13(8):784. doi: 10.3390/vetsci13080784 (PMC13517799; doi:10.3390/vetsci13080784)
Supplement: Supplementary file 1 [file vetsci-13-00784-s001.zip › Supplementary_Material_S4.pdf]

Supplementary Material S4

Illustrative Structure and Example Screens of the Prototype Online Animal Abuse Reporting Interface

This supplementary material provides an illustrative overview of the prototype online reporting interface developed for suspected animal abuse and animal welfare concerns in Hungary. It was prepared in response to the reviewer's request for a clearer presentation of how the interface was structured. The prototype is available in Hungarian at *https://allattudomanyi.moonshot-test.com*. The screenshots below are presented in their original Hungarian-language form, with English explanatory captions for international readers.

The interface was designed to guide the reporter through structured, observation-based questions rather than requiring a legal or veterinary classification in advance. Users first select the relevant reporting context, then proceed through pathway-specific questions covering the location, animals involved, observable condition, environmental circumstances, potential risk factors, optional photo upload, and reporter contact details. The examples shown here are illustrative and do not reproduce every possible answer option or all conditional branches of the live prototype.

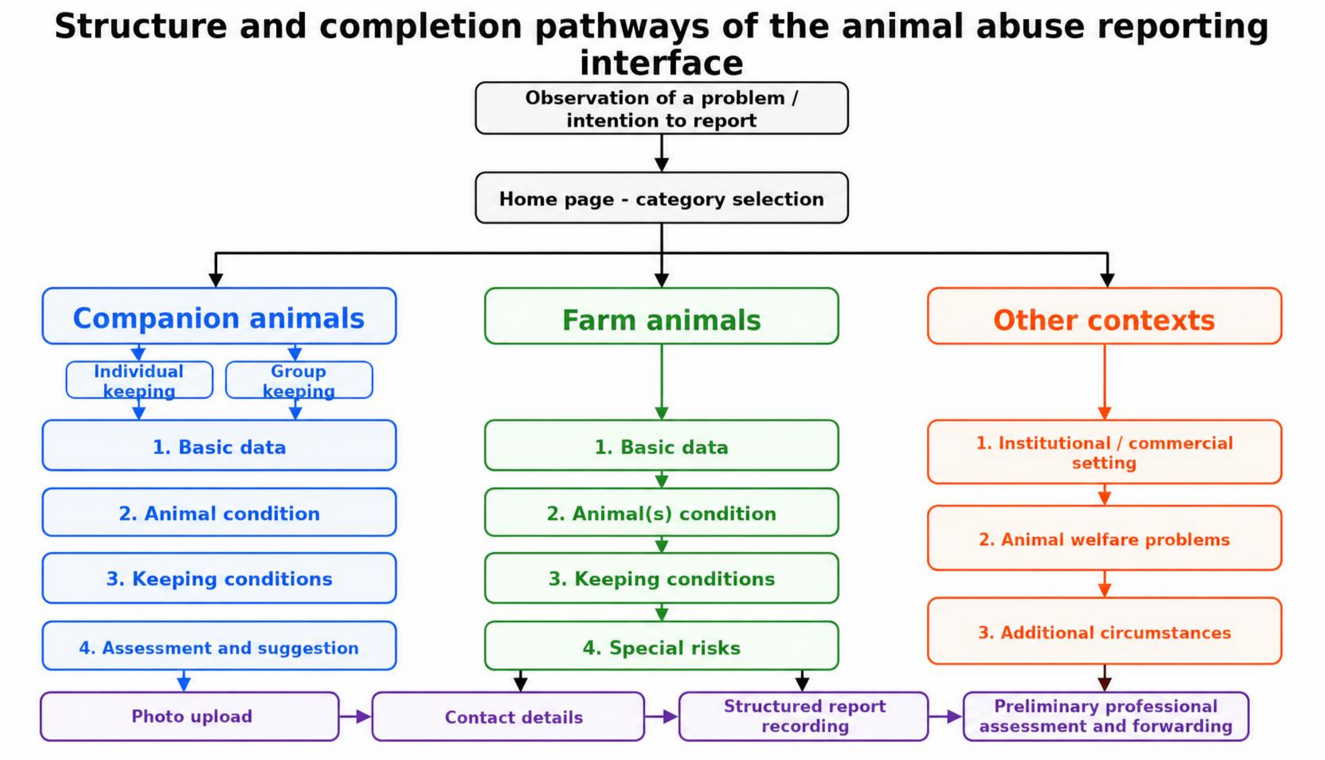

Figure S1. Conceptual overview of the reporting interface structure and completion pathways. The figure summarises the three main reporting contexts (companion animals, farm animals, and other contexts), their internal pathways, and the common downstream steps of photo upload, contact details, structured report recording, and preliminary professional review/forwarding.

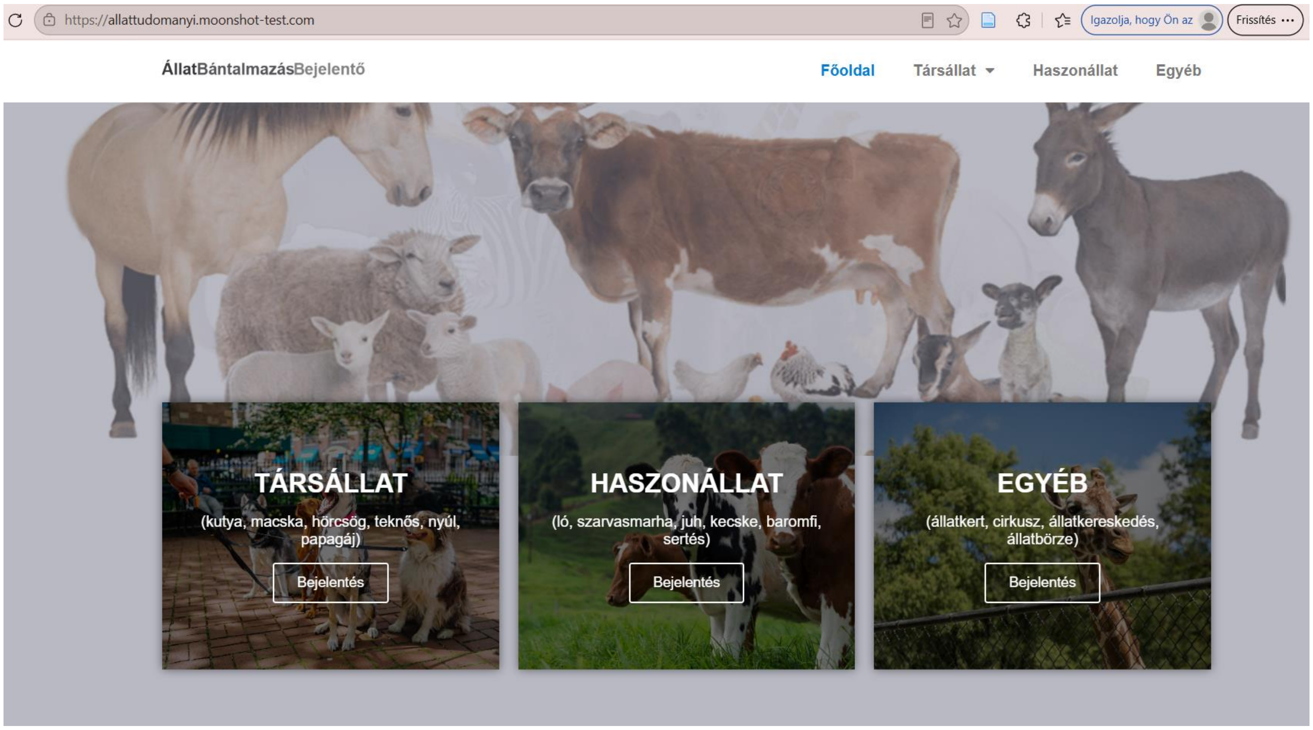

Figure S2. Homepage of the prototype interface. From the main page, users choose among three broad reporting contexts: "Társállat" (companion animals), "Haszonállat" (farm animals), and "Egyéb" (other contexts, such as zoos, circuses, animal trade, or animal markets).

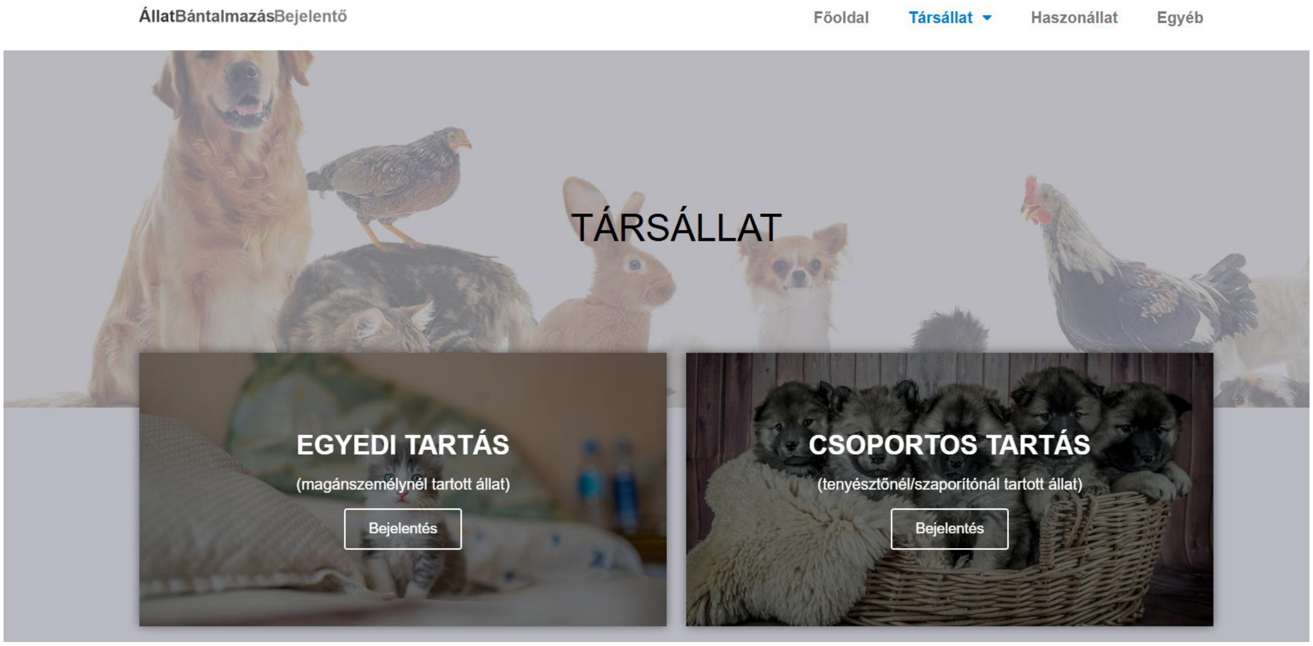

Figure S3. Companion animal category page. Within the "Társállat" (companion animal) reporting context, users choose between "Egyedi tartás" (individual keeping, typically privately kept animals) and "Csoportos tartás" (group keeping, such as breeder- or multiplier-kept animals).

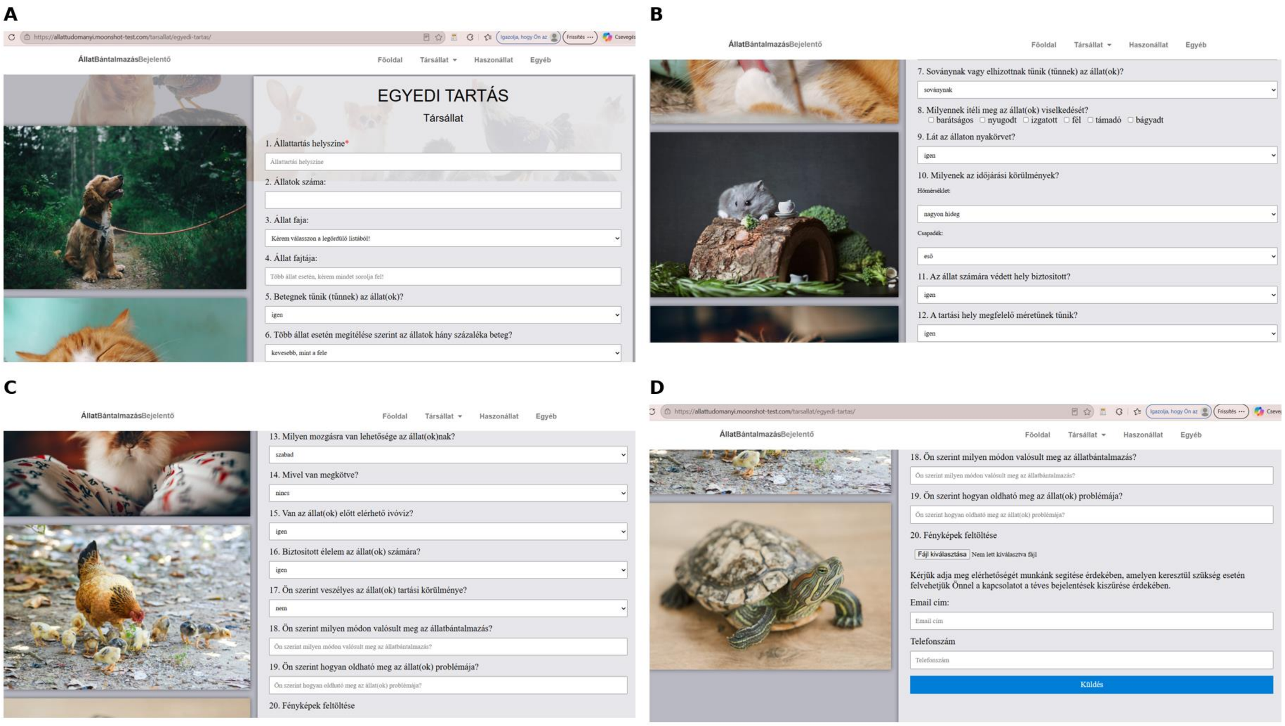

Figure S4. Example screenshots from the companion animal–individual keeping reporting form. Panel A shows the opening section with location, number of animals, species, breed, apparent illness, and the proportion of affected animals. Panel B shows observable condition and environmental questions, including body condition, behaviour, collar presence, weather, shelter, and housing adequacy. Panel C shows questions on movement, tethering, access to drinking water and food, danger, and the reporter's own assessment and proposed solution. Panel D shows the closing section with photo upload and optional contact information.

Table S4. English summary of the companion animal–individual keeping pathway

Module / section	Illustrative examples from the Hungarian prototype	Information collected	Purpose in the reporting process
1. Basic case information	"Állattartás helyszíne"; "Állatok száma"; "Állat faja"; "Állat fajtája"	Location of the keeping site; number of animals; species; breed/type or listed breeds when multiple animals are present	Defines the basic case context and helps identify the animal group involved.
2. Observable animal condition	"Betegnek tűnik…?"; "az állatok hány százaléka beteg?"; "Soványnak vagy elhízottnak tűnik"; "viselkedés"; "Lát az állaton nyakörvet?"	Perceived illness; proportion of affected animals; apparent body condition; observable behaviour; visible collar or restraint-related clue	Collects lay observations about the animal's apparent health, condition, and presentation without requiring diagnosis.
3. Environmental and housing conditions	"Milyenek az időjárási körülmények?"; "Védett hely biztosított?"; "A tartási hely megfelelő méretűnek tűnik?"; "Milyen mozgásra van	Weather conditions; shelter availability; apparent adequacy of enclosure size; freedom of movement	Documents contextual factors that may affect welfare and severity assessment.

	lehetősége?”		
4. Care-related resources and risk factors	“Mivel van megkötve?”; “Van az állat(ok) előtt elérhető ivóvíz?”; “Biztosított élelem?”; “veszélyes az állat(ok) tartási körülménye?”	Type of tethering if present; access to drinking water; access to food; perceived danger or hazardous circumstances	Captures essential husbandry factors and possible immediate risks.
5. Reporter interpretation and free-text comments	“Ön szerint milyen módon valósult meg az állatbántalmazás?”; “Ön szerint hogyan oldható meg az állat(ok) problémája?”	Reporter’s own description of the suspected problem and proposed solution	Allows additional context while preserving the structured core of the report.
6. Documentation and contact	“Fényképek feltöltése”; “Email cím”; “Telefonszám”	Optional image upload; reporter contact details	Supports evidentiary documentation and enables follow-up if clarification is needed.
7. Submission and downstream handling	“Küldés”	Final submission of the completed report	Forwards the structured report for preliminary professional review and possible further action.

Note. The prototype interface was developed in Hungarian. The wording shown in the screenshots reflects the Hungarian test version, whereas the table above provides an English-language summary for manuscript purposes. The prototype should be interpreted as a structured intake interface and not as a validated diagnostic, veterinary forensic, administrative, or criminal decision-making tool.
