# Supplementary material for: From Observation to Structured Reporting: Development and Preliminary Evaluation of an Online Animal Abuse Reporting System
Source: Vet Sci. 2026 Aug 5;13(8):784. doi: 10.3390/vetsci13080784 (PMC13517799; doi:10.3390/vetsci13080784)
Supplement: Supplementary file 1 [file vetsci-13-00784-s001.zip › Supplementary_Table_S1_Public_Questionnaire_1.pdf]

Supplementary Table S1. First Public Questionnaire

Public knowledge of animal protection procedures and reporting preferences

Participant information and consent statement

Participation in this anonymous questionnaire was voluntary. The questionnaire formed part of a scientific study on the reporting of suspected animal abuse, animal welfare concerns, and animal protection procedures in Hungary. The survey did not collect names, contact details, addresses, or other directly identifying personal information. Answers were analysed in aggregated form for scientific purposes, and individual respondents were not identifiable in any publication. Respondents could discontinue completion before submission. By submitting the completed questionnaire, respondents indicated consent to the anonymous use of their responses for scientific analysis and publication.

	**Question**	**Response format / answer options**
1	Age group	Single choice: 18–35 years; 36–65 years; over 65 years.
2	Gender	Single choice: female; male.
3	In your opinion, which persons or organisations are officially responsible for acting in animal protection cases related to animal abuse?	Multiple choice: police; notary/municipality; private veterinary practitioner; official veterinarian; animal welfare organisations; National Food Chain Safety Office; government office; I do not know.
4	In your opinion, which persons or organisations are officially responsible for acting in animal protection cases related to substandard animal keeping?	Multiple choice: police; notary/municipality; private veterinary practitioner; official veterinarian; animal welfare organisations; National Food Chain Safety Office; government office; I do not know.
5	If you have previously submitted a report in an animal protection case, did you find the reporting process complicated?	Single choice: yes; no. Conditional question for respondents with previous reporting experience.
6	If the reporting process were simpler, would you use this possibility?	Single choice: yes; no. Conditional question.
7	Which reporting option would you prefer?	Multiple choice: telephone; email; online reporting interface; application-based reporting; other/open response.
8	In your opinion, how could false or inaccurate reports be filtered out most effectively?	Multiple choice: guided questions; photograph upload; rapidly accessible reporting at the site of observation, for example online; other/open response.

Note: This document provides the English translation of the questionnaire items and response options for supplementary material purposes. The original questionnaires were administered in Hungarian.
