# Supplementary material for: From Observation to Structured Reporting: Development and Preliminary Evaluation of an Online Animal Abuse Reporting System
Source: Vet Sci. 2026 Aug 5;13(8):784. doi: 10.3390/vetsci13080784 (PMC13517799; doi:10.3390/vetsci13080784)
Supplement: Supplementary file 1 [file vetsci-13-00784-s001.zip › Supplementary_Table_S2_Public_Questionnaire_2.pdf]

Supplementary Table S2. Second Public Questionnaire

Public experiences with observing and reporting suspected animal abuse and substandard animal keeping

Participant information and consent statement

Participation in this anonymous questionnaire was voluntary. The questionnaire formed part of a scientific study on the reporting of suspected animal abuse, animal welfare concerns, and animal protection procedures in Hungary. The survey did not collect names, contact details, addresses, or other directly identifying personal information. Answers were analysed in aggregated form for scientific purposes, and individual respondents were not identifiable in any publication. Respondents could discontinue completion before submission. By submitting the completed questionnaire, respondents indicated consent to the anonymous use of their responses for scientific analysis and publication.

General questions and suspected animal abuse

	Question	Response format / answer options
1	Age group	Single choice: 18–35 years; 36–65 years; over 65 years.
2	Gender	Single choice: female; male; prefer not to answer.
3	In which county do you live?	Open-ended text response.
4	Have you personally observed animal abuse in the last three years, either in the street or anywhere in your immediate environment?	Single choice: yes; no.
5	If you observed animal abuse, what did you see?	Open-ended text response. Conditional question.
6	If you observed animal abuse, did you do anything?	Single choice or open response: spoke to the person but did not report it; spoke to the person and reported it; reported the case; no action; other/open response. Conditional question.
7	Why did you not report it?	Multiple choice: uncertainty about the reported person; uncertainty about the animal’s future; did not know where/to whom to report; fear of consequences; unable to prove the observation; not interested; other/open response. Conditional question.
8	How did you report the case?	Multiple choice: telephone; email; in person; online; application; other/open response. Conditional question.
9	To whom or where did you report the case?	Multiple choice or open response: police; notary; municipality; animal welfare organisation; National Food Chain Safety Office; district veterinary authority; public online platform, for example Facebook; other/open response. Conditional question.

Substandard animal keeping conditions

	Question	Response format / answer options
10	Have you personally observed an animal kept under substandard conditions in the last three years? Examples include permanent tethering, starvation, overfeeding, or exposure to extreme weather.	Single choice: yes; no.
11	If yes, what did you observe?	Open-ended text response. Conditional question.
12	Did you do anything?	Single choice or open response: spoke to the animal keeper but did not report it; spoke to the animal keeper and reported it; reported the case; no action; other/open response. Conditional question.
13	Why did you not report it?	Multiple choice: uncertainty about the reported person; uncertainty about the animal's future; did not know where/to whom to report; fear of consequences; unable to prove the observation; not interested; other/open response. Conditional question.
14	If you submitted a report, how did you report it?	Multiple choice: telephone; email; in person; online; application; other/open response. Conditional question.
15	Where or to whom did you submit the report?	Multiple choice or open response: police; notary; municipality; animal welfare organisation; National Food Chain Safety Office; district veterinary authority; public online platform, for example Facebook; other/open response. Conditional question.

Note: This document provides the English translation of the questionnaire items and response options for supplementary material purposes. The original questionnaires were administered in Hungarian.
