# Supplementary material for: From Observation to Structured Reporting: Development and Preliminary Evaluation of an Online Animal Abuse Reporting System
Source: Vet Sci. 2026 Aug 5;13(8):784. doi: 10.3390/vetsci13080784 (PMC13517799; doi:10.3390/vetsci13080784)
Supplement: Supplementary file 1 [file vetsci-13-00784-s001.zip › Supplementary_Table_S3_Professional_Questionnaire.pdf]

# Supplementary Table S3. Professional Questionnaire

*Professional experiences with animal protection reports and prototype-related needs*

**Participant information and consent statement**

Participation in this anonymous professional questionnaire was voluntary. The questionnaire formed part of a scientific study on the reporting and handling of suspected animal abuse and animal welfare cases in Hungary. The survey did not collect names, contact details, workplace addresses, or other directly identifying personal information. Answers were analysed in aggregated form for scientific purposes, and no individual respondent or institution was identifiable in any publication. Respondents could discontinue completion before submission. By submitting the completed questionnaire, respondents indicated consent to the anonymous use of their responses for scientific analysis and publication.

| | Question | Response format / answer options |
|---|---|---|
| 1 | What is your role or position in relation to animal abuse or animal protection cases? | Open-ended text response. |
| 2 | How do you usually receive reports from citizens? | Multiple choice: telephone; email; in person; online; application; I do not know; other/open response. |
| 3 | Approximately what percentage of reported cases are genuine animal protection cases, rather than ownership disputes or other non-animal-protection matters? | Single choice: 0–20%; 20–40%; 40–60%; 60–80%; 80–100%. |
| 4 | How emotionally burdensome is it to conduct an animal protection case? | Single choice or open response: case-dependent; emotionally burdensome; less burdensome due to routine; not emotionally burdensome; clients/reporters are the main burden; other/open response. |
| 5 | How competent do you feel in assessing the condition of the animal in an animal protection case? | Single choice: not competent, veterinarian needed; competent, but veterinarian also needed; competent, veterinarian not needed; not competent, but veterinarian not specifically needed. |
| 6 | Based on your experience, how long does it usually take to handle a case from the report until final closure? | Single choice: less than 1 month; 1–3 months; 3–6 months; 6–12 months; more than 12 months. |
| 7 | In your opinion, what could help accelerate the procedure? | Multiple choice: training; more competent actors; more staff dedicated to animal protection; faster/simpler reporting; legal amendment; state funding; public education on required report content; harmonisation/review of overlapping legal rules; other/open response. |
| 8 | Would it support your work if citizen reports in animal abuse and animal protection cases were submitted through a unified interface? | Single choice: yes; no. |

*Note: This document provides the English translation of the questionnaire items and response options for supplementary material purposes. The original questionnaires were administered in Hungarian.*
